# Enhancing maternal and newborn outcomes in Ghana: a comprehensive randomized controlled trial evaluation of obstetric triage effectiveness and midwives training

**DOI:** 10.1186/s12913-025-13132-7

**Published:** 2025-10-21

**Authors:** Antonella Bancalari, Julia Loh, Mary Eyram Ashinyo, Medge Owen, Britta Augsburg

**Affiliations:** 1 Institute for Fiscal Studies, London, UK; 2 Ghana Health Services, Accra, Ghana; 3 Department of Maternal and Child Health , Gillings School of Global Public Health, University of North Carolina at Chapel Hill, Chapel Hill, USA; 4 School of Medicine, Wake Forest University, Winston Salem, North Carlina USA

**Keywords:** Obstetric triage, Midwife knowledge and behavior, Maternal and neonatal outcomes

## Abstract

**Background:**

Ghana has made progress in maternal and newborn health, but significant challenges remain, with maternal mortality at 263 per 100,000 live births and neonatal mortality at 22.8 per 1,000 live births. The Obstetric Triage Implementation Package (OTIP), which includes rapid triage protocols and midwife-led peer training, aims to improve the quality of care in a context of scarce resources. This study evaluates the effectiveness of OTIP in improving maternal and neonatal outcomes and assesses the mechanisms behind the observed effects.

**Methods:**

A cluster randomized controlled trial will assess the impact of OTIP in 25 high-volume hospitals across Ghana during the final phase of its national roll-out. Hospitals will be randomized into early and late intervention groups. A complementary regression discontinuity analysis will assess the impact of OTIP at the national level. Primary outcomes include process improvements and maternal and neonatal outcomes. Secondary outcomes will assess midwives’ knowledge and attitudes. Data sources include primary surveys of 1,250 mother-newborn pairs and 750 midwives, and administrative records from the Ghana Health Service, Ministry of Health.

**Discussion:**

A rigorous evaluation of OTIP would be crucial, not only to assess the effectiveness of a program in which the Government of Ghana is already investing, but also to assess the potential applicability of this training model to other areas, and to contribute to the academic literature by filling gaps in our understanding of how different training methods can overcome barriers to the diffusion of new practices.

**Trial registration:**

Study protocols have been approved by the Ghana Health Service (GHS) Ethics Review Committee (GHS-ERC: 022/05/24). Trial registration number: ISRCTN15629047. Registered on 04/11/2024 while participant enrollment was ongoing, 10.1186/ISRCTN15629047. The study is hence retrospectively registered.

**Supplementary Information:**

The online version contains supplementary material available at 10.1186/s12913-025-13132-7.

## Background and rationale

Ghana has made considerable progress on key human capital indicators, such as reducing infant and child mortality rates, and has seen a significant rise in institutional deliveries over the past decade [1]. However, many hospitals still face shortages of critical resources needed to treat high-risk patients, while the growing workload negatively impacts the quality of care provided [2].

Ghana’s maternal mortality was 263 per 100,000 live births in 2020 [3] and neonatal mortality rate 22.8 per 1,000 live births, both remaining behind targets set by the United Nations Sustainable Development Goals [4].

The need to build the capacity of healthcare providers is widely acknowledged. Given the common challenges of low adherence to best practices and the limited fiscal space in government budgets, officials are emphasizing the need to better utilize existing resources. Peer-to-peer learning, supervision, and support—through feedback and mentoring—are seen as essential for maintaining quality.

The Obstetric Triage Implementation Package (OTIP) is designed to address these needs. OTIP consists of a 1-week on-site training on clinical knowledge to quickly assess and prioritize the care of pregnant women, with the goal of promptly and accurately identifying the severity of a patient’s condition, determine the appropriate level of care, and ensure that those with the most critical needs receive immediate attention.

A key innovation of OTIP is the designation of up to ten midwives per hospital as ‘Champions’—those selected to attend onsite training and lead the implementation of the new triage protocol. These Champions are responsible for training their peers, as well as monitoring and motivating the adoption of the protocol among colleagues and setting up and maintaining a triage room.

Pilot studies have demonstrated significant reductions in patient waiting times, highlighting the potential to improve both the efficiency and quality of service delivery [5, 6], consistent with evidence from on-the-job training by more experienced or skilled workers in the education sector [7, 8]. However, the scalability of OTIP and its effectiveness remain uncertain. Conducting rigorous research on its implementation at scale is critical, as promising interventions often face challenges in scaling up [9].

A rigorous evaluation of OTIP Champions would be crucial not only for assessing the potential application of this training model to other areas of GHS, such as essential care for small babies and kangaroo mother care, but also for contributing to the academic literature by addressing gaps in our understanding of how different training methods can overcome barriers to the diffusion of new practices [10].

### Objectives

The project’s key research questions are as follows:

1. What is the impact of OTIP on the quality of service during labor and delivery, as well as on maternal and neonatal survival, and on neonatal health outcomes? 
2. To what extent does OTIP affect clinical knowledge and midwives’ attitudes? 

### Methods and trial design

**Fig. 1:**
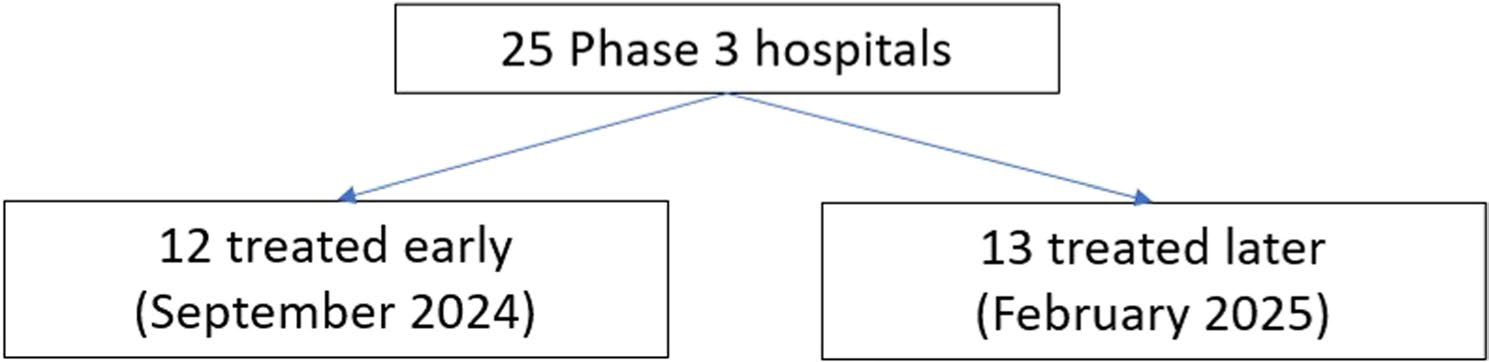
Randomization flow Diagram

This study uses a cluster randomized controlled trial (cRCT) to evaluate the impacts of the introduction of the OTIP Champions program across high-volume hospitals in the final phase of its nation-wide rollout. The cRCT will be integrated into the final phase of the national rollout of the OTIP training program, covering the Central, Greater Accra and Western regions. 12 of the 25 hospitals are randomly assigned to receive OTIP training in September 2024, and the remaining 13 receive OTIP approximately 4 months later. Figure 1 is the design flow diagram. SPIRIT reporting guideline were used [11].

#### Study setting

The Government of Ghana, through Ghana Health Services, is rolling out OTIP to all high-volume hospitals (defined as hospitals with more than 1,200 births in 2022) in different regions of the country. In 2023, the average total number of deliveries in these high-density hospitals was 2,146. The average maternal mortality rate was 110 per 100,000 live births, and the average neonatal mortality rate was 8.21 per 1,000 live births. In hospitals across Ghana, the average total number of deliveries in 2023 was lower, at 1,688. The average maternal mortality rate was slightly higher at 126 per 100,000 live births, while the average neonatal mortality rate was slightly lower at 7.7 per 1,000 live births.

#### Eligibility criteria

The cRCT will focus on the 25 hospitals that will receive the OTIP program during the final phase of the national roll-out, which has been planned in three phases (see Supplementary Materials S.3 for names of study sites). The first two phases of the roll-out were completed by April 2024. The third and final phase of the rollout was started in September 2024, covering all eligible hospitals, 25 in total, in Greater Accra, Central and Western regions.

Within each study hospital, target participants will be mothers that delivered in the hospital within the last two months prior to data collection. They will not be selected if they are facing post-birth complications, or if they are below the age of 18 and a guardian is not present. Data from mothers recruited in January 2024 will be the basis for the impact evaluation. We in addition collect information from mothers who were present prior to intervention implementation. These will however not be exposed to the OTIP intervention.

We will further conduct interviews with individuals who will perform the intervention, e.g. midwives. We will interview those that are selected to be OTIP champions, and a random sample of non-champion midwives on permanent contracts in the maternity department, including those in charge of wards and clinics. We will also administer online surveys to all the midwives on permanent contracts working in each hospital to collect data on clinical knowledge (1,338 midwives in total).

#### Intervention

The OTIP Champions Program, developed by the non-governmental organization Kybele, Inc. in partnership with GHS, consists of a 1-week on-site training on clinical knowledge and a new triage protocol to quickly assess (in less than 10 min since arrival) and prioritize the care of pregnant women (using color-coded wristbands), with the goal of promptly and accurately identifying the severity of a patient’s condition, determine the appropriate level of care, and ensure that those with the most critical needs receive immediate attention. At the core of its design, OTIP integrates rapid and accurate patient assessment and care planning as a routine part of midwives’ practice [12].

**Fig. 2:**
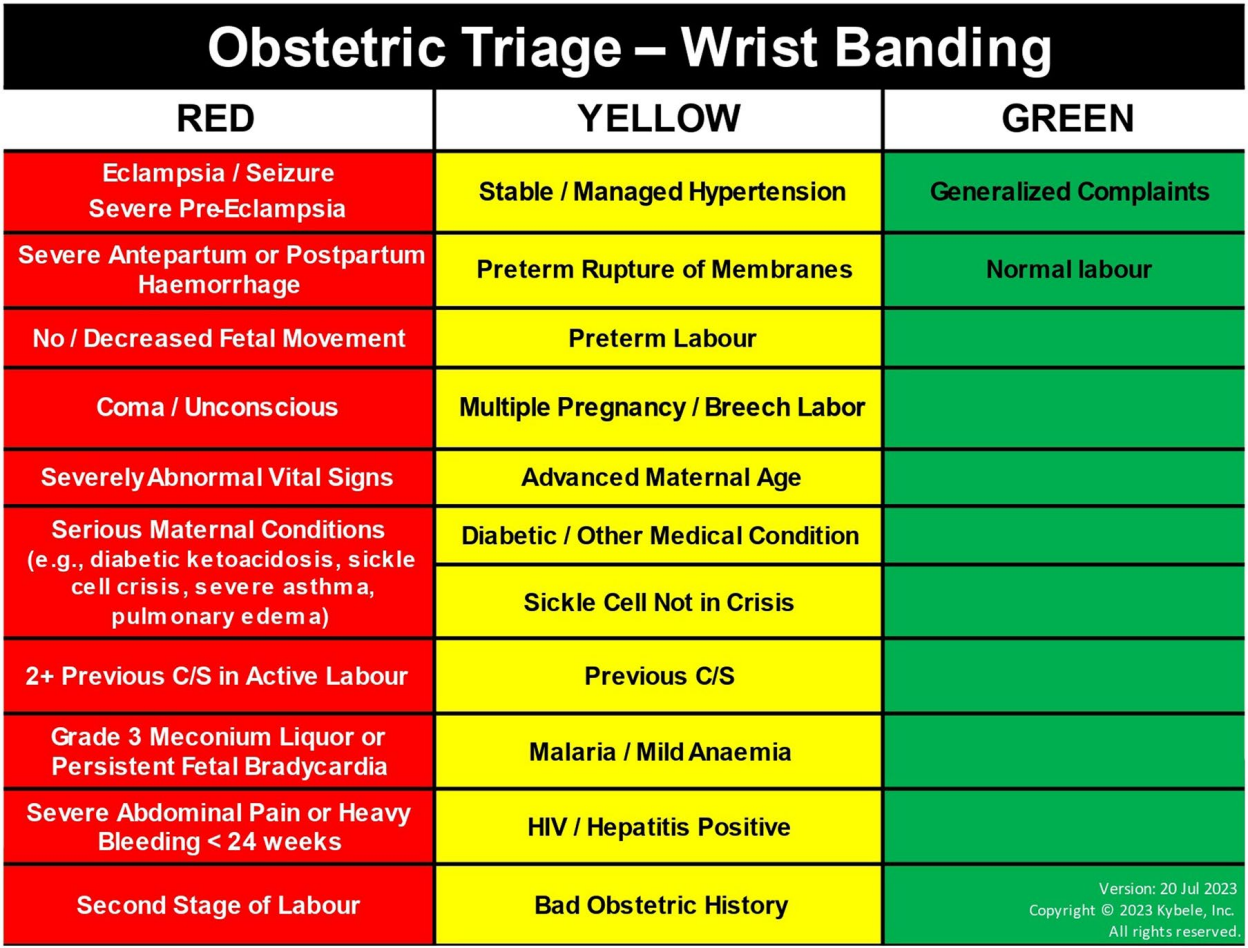
OTIP risk classification

The intervention also introduces a dedicated obstetric triage area in hospitals, where midwives systematically evaluate the patient’s obstetric and medical history, vital signs and labor progress on a standardized triage assessment sheet, resulting in a categorization of high, intermediate, or low risk, and the application of a corresponding color-coded patient wristband. See Fig. 2 for more details on categorization. A care plan is developed and documented based on the diagnosis and risk status. High-risk pregnancies require immediate intervention, with a doctor involved, intermediate-risk cases require careful and frequent monitoring, and low-risk cases proceed to normal delivery with the assistance of a midwife.

With the support of Kybele, the national trainers–GHS healthcare staff (OB-GYN doctors and midwives) who were part of the pilot OTIP program— train the champion midwives in their own hospital premises. The first day of training covers motivation on the job, clinical knowledge, as well as theoretical and practical session on how to use the new obstetric package (i.e., triage assessment forms, risk acuity charts and color-coded bands), and how to develop and implement care plans based on the diagnosis and risk status of patients.

The second day of training covers a module on monitoring (i.e., record keeping) and leading (i.e., how to deal with change opposition) OTIP’s implementation. The next two days consist of formal training sessions led by the Champions, where they teach their own peers the content of the first day of training with support and guidance from the national trainers. The final day consists of setting up the new triage area. After hospital management identifies an appropriate area, Champions help to clean, order, and arrange the necessary equipment (plus vital signs monitoring tools donated by Kybele), assessment surveys and forms, as well as color-coded bands. Champions also select among themselves who the triage in-charge will be.

The selection of Champions among midwives is conducted by either hospital management or fellow midwives. They are selected based on criteria that capture the extent to which midwives have leadership and teaching abilities. The exact content was developed from Kybele’s prior experience in Ghanaian hospitals.

#### Outcomes

We consider two sets of primary outcomes:

- Process variables: Representing the actual medical care received by mothers and their new-borns (e.g., time until initial assessment; assessment received upon arrival; doctor intervened if complications during labour and delivery at the hospital; postnatal checks).
- Maternal and neonatal outcome variables: Including the health of pregnant women and new-borns (e.g. maternal and neonatal mortality; complications during labour and delivery at the hospital; APGAR scores).

To examine mechanisms behind potential impacts of OTIP on the primary outcomes, we further consider secondary outcomes:

- Hospital staff outcomes: Such as improved knowledge and midwives’ attitudes capturing their perceptions of autonomy, empowerment and motivation.

#### Sample size and power

The cRCT will focus on 25 OTIP hospitals. We aim to interview on average 30 hospital staff per hospital and 50 mother-newborn pairs. In total, the sample for the cRCT will be 750 staff, and 1250 mother/newborns per data collection round.

We calculate minimum detectable effects (MDEs) using the following formula [13]:

1 $$MDE\left(\frac{\left(t_{\alpha/2}+t_\beta\right)^2\sigma^2(mp\left(1-R_c^2\right)+\left(1-p\right)\left(1-R_p^2\right)}n\right)^\frac12$$

$\:\alpha\:$ denotes the significance level, $\:\beta\:$ power, $\:\rho\:$ unconditional ICC, $\:t$ is the critical values of the t-distribution, $\:\sigma\:$ is the standard deviation of the outcome,$\:\:n$ is midwives/mothers per arm, $\:m$ is midwives/mothers per cluster, $\:{R}_{c}^{2}$ is proportion of cluster level variance component explained by covariates and $\:{R}_{p}^{2}$ is the individual equivalent. One can interpret the effect of $\:{R}_{c}^{2}$ and $\:{R}_{p}^{2}$ as altering the “variance inflation factor” of the power calculation– when they equal zero the formula reduces to the standard cRCT one, applicable in our case.

To calculate the minimum detectable effects (MDEs), we assume a significance level ($\:\alpha\:$) of 0.05 and power ($\:\beta\:$) of 0.8 using two-sided tests.

To estimate intra-cluster correlations (ICCs) for the outcomes we measure, we reference the 2005 WHO Global Survey on Maternal and Perinatal Health [14], which presents ICCs that were obtained from 97,095 pregnancies and 98,072 births taking place in a representative sample of 120 hospitals in eight Latin American countries:

- Process variables: ICCs range from a minimum of 0.0003 to a maximum of 0.563, with a median of 0.067.
- Maternal outcome variables: Median ICC of 0.011 (interquartile range 0.007–0.037).
- Neonatal outcomes: Median ICC of 0.054 (interquartile range 0.013–0.075).

For hospital staff outcomes, we use data collected by colleagues on midwives in Nigeria to get an idea of hospital staff outcomes. The data provide indices that capture emotional exhaustion, accomplishment, and depersonalization (based on the Maslach Burnout Inventory).

- Hospital staff outcomes: ICCs of 0.07 for emotional exhaustion, 0.12 for accomplishment, and 0.07 for depersonalization.

As we will collect baseline data, we will be able to gain precision, allowing us to reduce ICC. We therefore assume ICCs of between 0.01 and 0.3 for process outcomes, 0.01 for maternal and neonatal outcomes, and no more than 0.1 for hospital staff outcomes.

Table 1 provides MDEs for all outcome groups. We note that since we assume that all outcomes considered will be continuous normally distributed variables standardized to have mean zero and standard deviation of one relative to the control group, the estimated MDE’s do not vary by outcomes, but rather by sample size we have for each outcome group.

**Table 1:** Minimum detectable effects (standard deviations)

Outcome	MDE for different ICCs		
	0.01	0.1	0.3
Process (1,250)	0.196	0.390	0.637
Mother/child (1,250)	0.196	0.390	
Midwife level (750)	0.236	0.410	

For process variables, our RCT sample yields MDEs between 0.196 and 0.64 for two-sided tests. For maternal and newborn outcomes, the MDE is between 0.196 and 0.39. For hospital staff outcomes, the expected MDE is between 0.36 and 0.41.

#### Participant timeline

**Fig. 3:**
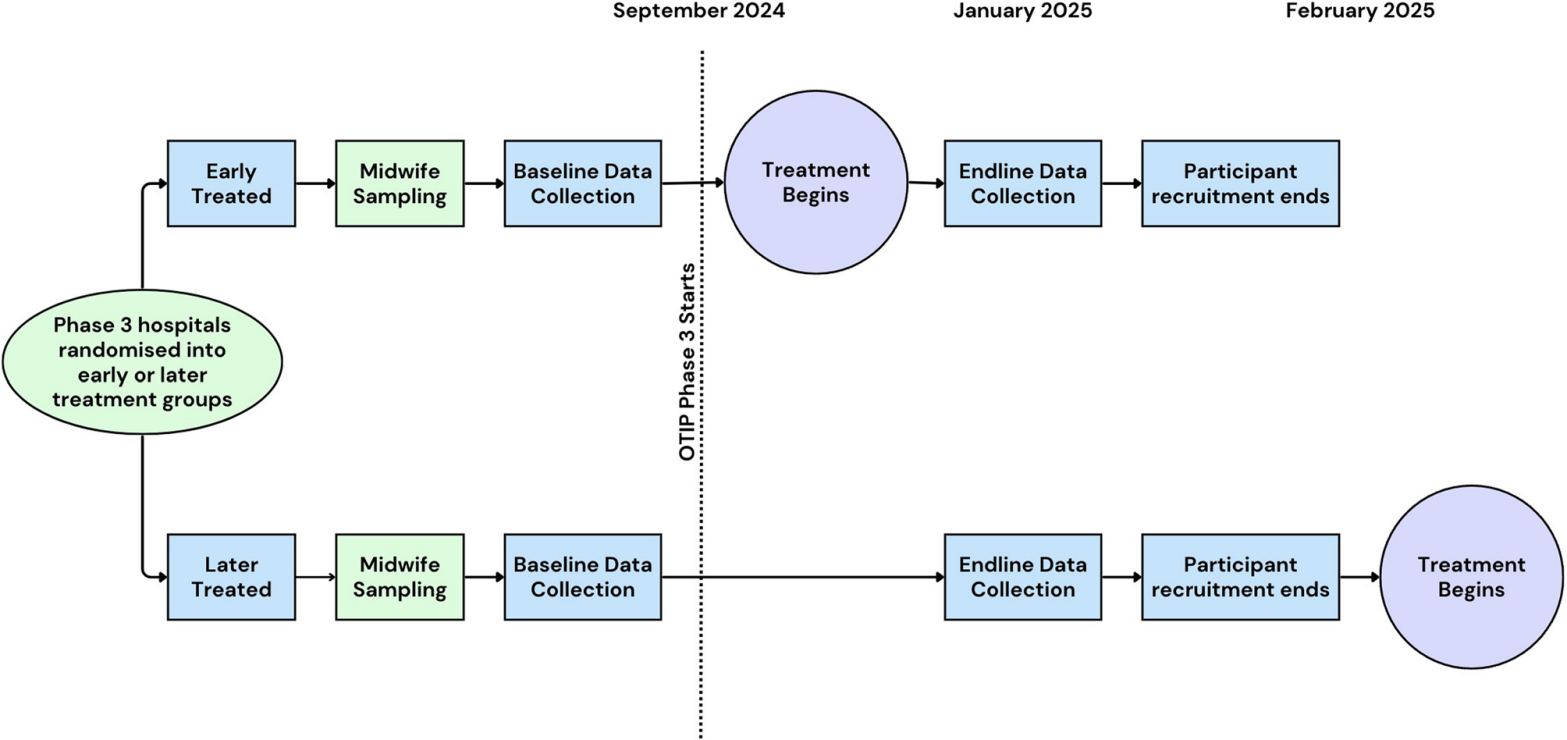
Participant Timeline

Hospitals were randomized into early and late treated in July 2024. Pre-intervention implementation data was collected in September 2024. The first set of OTIP trainings took place in October 2024, the second will be conducted in February 2025. Data collection for primary outcome analysis will be conducted in January 2025. Mothers for the impact analysis will be recruited from early January 2025 and data collection is expected to take one month. The participant timeline is presented in Fig. 3.

#### Sample size, sampling, and recruitment

The total targeted sample size for the cRCT will be in-person interviews with 750 staff, and 1,250 patients (mothers) per data collection round. In addition to the in-person survey we will administer online surveys to all the midwives on permanent contracts working in each hospital to collect data on clinical knowledge (1,338 midwives in total).

The patients target sample sizes proportional to each hospital’s delivery caseload in August-October 2023. This results in a minimum sample size of 35 and a maximum sample size of 65 per hospital. The inclusion criteria will be those who delivered in the hospital within the last two months, primarily from labor wards, postnatal wards, and postnatal care clinics. This eligibility criteria implies that in each survey round, we will interview a new cross section of 1,250 mothers. For mothers 18 years or younger, interviews will only be conducted if a guardian is present and provides consent in addition to the mother doing so herself. Mothers will only be approached for interview if not in distress due to, for example, having lost their child during labor. We will cross-check with hospital staff whether mothers can be approached or not.

The sample of midwives for these interviews will consist of the 10 midwives nominated by management or their peers to become champions (of whom 7 will ultimately be selected for training) and a random sample of 20 other midwives with permanent contracts in the maternity department, resulting in a total midwife sample of 750 midwives. We will not interview any students or staff on temporary contracts in the hospital. We aim to interview the same set of midwives throughout the study.

#### Assignment of interventions

Randomization was conducted using Stata version 16 by researchers at the IFS. The randomization determined whether a hospital would receive the intervention earlier or later. The decision whether a patient is assigned to the intervention or control group therefore depends on the timing of inclusion. By end February 2025 all hospitals will have received the intervention and hence by then every patient will be included in treatment. There is no blinding. Inclusion in the study for assessment of intervention effectiveness will be completed by the end of January 2025.

#### Data collection and management

We will rely on two main sources of data. For the cRCT, we will mainly rely on primary collected data from patients (mothers), and from midwives through in-person interviews. To promote data quality, all enumerators attend a week-long training session before data collection. This includes classroom-based training as well as training in hospital settings. It also covers how informed consent should be obtained, implying that informed consent will be acquired by well-trained and vetted data collection staff before the commencement of any data collection. Lead enumerators were also involved in two weeks of extensive piloting of the survey instruments.

For the regression discontinuity design, we will utilize the District Health Information Management System II (DIMS II), an electronic platform managed by GHS to collect, record, and analyze health service data nationwide. We will use key indicators such as deliveries and mortality for mother and new-born pairs at the hospital and monthly level from before, during and after OTIP implementation.

Administrative records and collected data will be recorded electronically using password-protected laptops. Once data collection is completed, all data will be promptly uploaded to an encrypted folder and/or deleted from the laptops. Access to the encrypted folder will be restricted to the research team, with participants’ identities anonymized in any reports derived from the research.

Once results are published, an anonymized version of the dataset will be released publicly without any personally identifying information.

The project is compliant with the Ghana Data Protection Act 2012 Act 846, and the UK Data Protection Act 1998.

For further details on data management: please refer to the project’s management registration: https://ifs.org.uk/sites/default/files/2024-11/Data-protection-V2.pdf. Data availability is outlined in online supplemental material S2.

#### Statistical methods

For our process variables, we will estimate the intent-to-treat effect of the program. Our design is a cluster randomized controlled trial stratified by region.

We estimate the impact of OTIP on process and maternal and neonatal outcomes by estimating the following specification for the outcome *Y* of mother or baby *i* in hospital *j* in region *r* at time *t*:

2 $$Y_{ij}=\beta\;OTIP_j+\delta\;X_{ij}+Y_r+\epsilon_{ij}$$.

Where $\:OTI{P}_{j}$ indicates if a hospital is allocated to receive OTIP early, $\:{\varvec{X}}_{ij}$ is a vector of controls for hospital characteristics selected by machine learning methods, and $\:{\gamma\:}_{r}$ are region fixed effects, as we stratified our randomisation by region to ensure balance in earlier and later treated hospitals in each region.

To increase statistical power, we will leverage the panel nature of our data and use baseline values of the outcomes in our analysis. For observations with values collected at baseline and follow-up, we can use an ANCOVA design. For observations without baseline values, we can use a difference-in-differences specification, exploiting variation across early- and later-treated hospitals, before and after the introduction of OTIP in early-treated.

To investigate mechanisms behind the observed impacts, we will use the same design with hospital staff outcomes to understand whether any impacts are driven by changes in staff outcomes due to OTIIP, such as midwives’ clinical knowledge and attitudes.

Moreover, for outcomes with low serial correlation, we will follow [15] and pool the multiple follow-up measurements to average out noise and increase power.

Due to the small number of clusters (25), we will present a wild-bootstrap p-value that is robust to small numbers of clusters [16]. We will additionally adjust for multiple hypothesis testing by family of outcome. Lastly, we will test for balance using baseline data for outcomes for hospital, midwives and patients’ characteristics. We will also test if attrition is uncorrelated to treatment allocation, and in case of non-random attrition on treatment status, we will estimate our main specification using inverse probability weighting.

#### Interim analyses

We plan to analyze descriptively the data collected at baseline to check for balances in baseline characteristics of samples from all treatment arms. This will be done in December 2025. We will also provide descriptive analysis on the pre-existing quality of care received by mothers in maternity departments of our study hospitals, as well as characteristics of midwife champions selected. These descriptive analyses will feature in a baseline report that will be made publicly available.

#### Methods for additional analyses

We will also leverage the fact that the OTIP Champions Program was implemented at scale in Ghana to complement our cRCT with an approach that allows us to look at mortality impacts. We will compare outcomes for hospitals that received OTIP with those that were not part of the program. Because the former set of hospitals might differ from the latter set in unobservable ways, we will use a regression discontinuity design (RDD). The intuition of RDD is that if OTIP affects outcomes, we would observe abrupt jumps in outcomes at a pre-determined eligibility threshold. Leveraging the arbitrary cutoff of 1,200 deliveries in 2022 for eligibility into the OTIP program, which can be deemed as good as random, we can robustly estimate impacts for all hospitals around the threshold.

The key assumption of the RDD strategy is that subject to no jumps in other unrelated characteristics, the jump in eligibility makes hospitals just above and just below the threshold comparable, in all characteristics, except in outcomes affected by OTIP. This key assumption can be tested by examining how variables unrelated to OTIP (such as A&E admissions) behave around the threshold.

To estimate the impacts of OTIP using RDD, we use the following specification for hospital$\:\:i$:

$$Y_i=\alpha+\beta\;del_i+\gamma\;Above_i+\epsilon_i$$

Where $\:del$ is the number of annual deliveries (the running variable) in hospital$\:\:i$ in 2022 and $\:Above$ is an indicator for whether hospital$\:\:i$ had more than 1,200 deliveries in 2022.

Our estimate of interest will be $\:\gamma\:$, which is the estimate for the difference in the outcome of interest attributed to OTIP. $\:{Y}_{i}$ represents the mortality rate during a period (monthly or annual). As a placebo test, we will present effects in mortality for the outcomes measured before the nationwide roll-out of OTIP in Ghana.

As the estimate of $\:\gamma\:$ is dependent on the functional form of the data, that is, how $\:Y$ changes with $\:{del}_{i}$, we will explore second order polynomials, if necessary, to get a better estimate of $\:\gamma\:$.

A key aspect of the RDD is defining a window of analysis around the OTIP eligibility threshold in the running variable (2022 deliveries). We will explore optimal bandwidths for this analysis.

Furthermore, the data gathered will be able to answer more scientific questions than outlined in this protocol. The study team expects to conduct and publish additional analyses.

#### Stopping rules

While there is always a risk of unintended consequences in all types of trials, in this sort of intervention such a risk is minimal. However, if there is any clear evidence of harm, then the study will halt under international ethnic guidelines for medical research. The strong partnership with GHS-MoH and Kybele, and the fact that this study was tailored to their needs ensures the sustainability of this study. The OTIP program, piloted and implemented in Ghanaian hospitals by Kybele since 2012, has shown resilience across different governments, offering strong assurance of its continuation.

#### Oversight and monitoring

Intervention implementation is managed by GHS and Kybele. The research team is in regular contact with the intervention management team, attending monthly update meetings and having ad-hoc calls where necessary. Data collection activities are conducted by the data collection company Data Pivot, in close collaboration with the research team. During data collection activities the teams have daily contacts, in-person and online. There is a low risk of data misuse. The P and Co-PIs have oversight of the research project.

#### Ethics and dissemination

The study is overseen by the Ghana Health Service Ethics Review Committee (Approval number GHS-ERC:022/05/24). Consideration will be given to potentially vulnerable people or groups, such as minors, and informed consent will be acquired by well-trained and vetted data collection staff before the commencement of any data collection. Moreover, patients can stop participating at any time without providing a reason. Consent forms are provided in Supplementary Materials S.1. Important protocol modifications will be communicated to all relevant parties.

Data will be collected, handled, transferred and secured in a manner which aligns with international best practice. The IFS information security management system is ISO27001 compliant.

We will publish findings through (a) top academic journals; (b) presentations at conferences in Ghana, the USA and Europe; (c) publication of high-impact policy briefs for the wider public; and (d) partner’s websites and social media. We will publish findings through (a) top academic journals; (b) presentations at conferences in Ghana, the USA and Europe; (c) publication of high-impact policy briefs for the wider public; and (d) partner’s websites and social media.

Whilst there is always a risk of unintended consequences in all types of trials, in this sort of intervention such a risk is minimal. However, if there is any clear evidence of harm then the study will halt under international ethical guidelines for medical research.

## Discussion

This trial assesses the effect of a programme previously demonstrated to be effective in a small pilot implementation now being brought to scale nationally by the Government of Ghana.

Scaling up interventions is often challenging; this study evaluates whether a scalable design can also be effective.

A challenge in this study is a relatively small number of randomization units and measurement of service quality.

## Supplementary Information

Supplementary Material 1.

## Data Availability

No data are available. The data collected in the study will be made publicly available following the publication of the primary results from the trials.

## Abbreviations

cRCT: Cluster randomized controlled trial
GHS: Ghana Health Service
ICC: Intra-cluster correlation
IFS: Institute for Fiscal Studies
MDE: Minimum detectable effects
OTIP: Obstetric Triage Implementation Package
RDD: Regression discontinuity design
