# Supplementary material for: Enhancing maternal and newborn outcomes in Ghana: a comprehensive randomized controlled trial evaluation of obstetric triage effectiveness and midwives training
Source: BMC Health Serv Res. 2025 Oct 21;25:1386. doi: 10.1186/s12913-025-13132-7 (PMC12542103; doi:10.1186/s12913-025-13132-7)
Supplement: Supplementary file 1 — Supplementary Material 1. [file 12913_2025_13132_MOESM1_ESM.docx]

**SUPPLEMENTARY MATERIALS**

[Enhancing Maternal and Newborn Outcomes in Ghana: A Comprehensive Randomized Controlled Trial Evaluation of Obstetric Triage Effectiveness and Midwives Training]

**S.1 Model consent forms:**

**INFORMED CONSENT FOR MOTHER/PRIMARY CAREGIVER**

**Introduction**

Good day, my name is [NAME OF ENUMERATOR]. I have come from DataPivot Ghana, which is a local data collection company. DataPivot is working in collaboration with researchers at the Institute for Fiscal Studies, University of Cape Coast, and Ghana Health Services.

**PART 1: INFORMATION SHEET**

**Background and purpose of the research:**

The study will contribute to our understanding of how to improve quality of early childhood service delivery. The focus is on improving the birth process, survival, child developmnt and the impacts such improvements have on maternal and child outcomes in the longer-term.

**Nature of the research**

We are asking all mothers who give birth in hospitals across Ghana to take part in this study. This is why you have been chosen. We will talk to these mothers like you, as well as midwives and other hospital staff in hospitals across Ghana. The results of this research will be shared with GHS and the Ministry of Health to understand how they can better design policies to improve the health and development of young children.

**Participant involvement:**

We will give you information to help you decide whether you would like to take part in the study. There may be some words that you do not understand. Please ask me to stop as we go through the information, and I will take time to explain.

*What is involved?* Today, we will take some personal information from you, such as your name and contact details, and ask you about your experience of this hospital, your thoughts and feelings. We will also ask for your consent to access information the hospital holds about you (such as the time of your arrival) and to access information about future postnatal check-ups if you chose to attend. We will provide more details on the type of data we aim to collect further below, when we also explaining how we use and look after your information. We might also show you a short video at the end of the interview. Once all information provided is clear to you, we will ask you for your consent to participate.

*Duration?* We expect today’s interview to take around thirty to fourty minutes once consent is provided. If you take part, we will also ask you for permission to contact you again in the future. This will be either to ask questions and/or to provide some information about child development. If we do contact you in future to ask for this information, we will ask you for your consent again.

*Right to Refuse or Withdraw:* Participation in this study is voluntary and you may choose not to participate. If you decide not to participate or to withdraw, there will be no repercussions. You can withdraw your consent from the study at any time, either during this survey or after. No data will be submitted without your explicit consent, which is gathered at the beginning and end of the survey. If, in the future, you wish to withdraw your details from the survey or withdraw your consent, you can do so at any time by contacting us at datapivotgh@gmail.com.

*Benefits of taking part in this research:* There are no direct benefits to you from participating in the survey. However, the information you would provide will help us design effective training programs for service delivery to (expecting) parents.

*Disadvantages and/or risks of taking part in the study:* The collection of your information will not put you or your child at risk. All those involved in data collection are aware that if anything untoward happens while they are conducting interviews, either in their interaction with research participants or in relation to the information they are given, they will report it up the chain of command. Such reporting means that OPM senior management that are based back at Head Office are quickly made aware of anything that is unexpected that may have a detrimental effect on the research participants. Senior management will then quickly decide how to mitigate any such unexpected complications or incidental findings. Additionally, please note that in the case the child is in immediate danger, midwives on duty will be called.

*Confidentiality:* All information we collect is strictly confidential and will only ever be used for academic research purposes. Only members of the research team will be able to access information that identifies you and it will only be used for academic research purposes either now or in the future.  Your information may be used by other researchers outside the team but all information that personally identifies you will be removed before it is shared. We provide more information about how we handle your personal data further below.

*Outcome and feedback:* A summary report of key findings will be made publicly available on the following websites: [www.ifs.org.uk](http://www.ifs.org.uk/), <https://www.opml.co.uk/>, and the Thrive project website: <https://www.opml.co.uk/projects/thrive-early-childhood-development>

*Funding information:* The research is funded by the Foreign, Commonwealth & Development Office (FCDO), UK, under contract reference PO10087.

*Provision of Information and Consent for participants:* We will give you a copy of this information sheet and the following consent form for you to keep after it has been signed or thumb printed.

**Contact information:**

If you have a question later that you didn't think of now, you can contact DataPivot and [datapivotgh@gmail.com](mailto:datapivotgh@gmail.com). If you want to get further clarification on ethical issues and your rights as participants you can contact Nana Abena Apatu on 0503539896 or at ethics.research@ghs.gov.gh. If there is any part of this explanation that you do not understand, you should ask before signing. For English speakers, you can also call +44 (0)207 291 4800, and ask for the Principal Investigator of this research: Dr. Britta Augsburg (Associate Director, The Institute for Fiscal Studies) or ask for Zoe Oldfield (Data Protection Officer (DPO), The Institute for Fiscal Studies). You can also contact both on e-mail ([britta_a@ifs.org.uk](mailto:britta_a@ifs.org.uk) and [zoe_o@ifs.org.uk](mailto:zoe_o@ifs.org.uk), respectively).

**PART 2: PRIVACY NOTICE**

Before asking for your consent, we will now read the privacy notice to you. Because the research team are based in the UK, your information is controlled by strict data protection laws. This privacy notice is a legal document and contains some complex language so please ask me if you do not understand something.

**PRIVACY NOTICE FOR MOTHER/PRIMARY CAREGIVER**

**What is a privacy notice?**

A privacy notice is an important document which tells you how we will use and look after any information that we collect about you and your child. This privacy notice contains all the information that we are required to give you under the UK General Data Protection Regulation (UK GDPR).

**Who are we?**

There are key organisations involved and responsible in the processing and storing of your information. These are the Institute for Fiscal Studies (IFS), Oxford Policy Management (OPM), the University of Cape Coast (UCC), and DataPivot. Our contact details can be found the end of this privacy notice.

**Who is responsible for looking after the information you have about me?**

“Data controller” is a legal phrase, which tells you who makes the decisions about how your information will be used and looked after. The IFS and OPM are the data controllers of the information collected as part of this study. This means that IFS and OPM are independently responsible for processing and looking after your information whilst the data are being collected and analysed by the team.  DataPivot will be responsible for carrying out the interviews with you and transferring the information to us and the University of Cape Coast will be supporting the work that DataPivot does. Because they act only on the instructions of IFS and OPM, DataPivot and UCC are data processors of your information.

**What information do we collect about you?**

The information that we collect, use and store will include personal information about you. Some of this information will be given to us by you and some of this will be given to us by GHS.

The information that we will ask you to tell us will include:

- Your name and contact details,
- The name and contact details of a relative, your husband, partner, or other person you nominate,
- Information about yourself, such as your age, race or ethnicity, whether you had other children in the past, your employment status,
- Information about your experience, thoughts, feelings, and behavior that you share with us as part of the questionnaires,
- The names and details of your community health practice.

We will also get some information that the hospital holds about you and your baby, such as:

- The time when you arrived in the hospital,
- Whether you received any medications,
- Tests given to your baby just after birth such as its heart rate, muscle tone, and other signs to see if extra medical care or emergency care is needed.

We will further ask you for your consent to access information collected during postnatal check-ups until your child is three years old. This information will include:

- How you feel,
- your blood pressure,
- your child’s height and weight
- it’s heart rate, muscle tone,
- abnormalities (if any),
- vaccinations,
- results of hearing screening.

**How do we use your information?**

We will use the information you give us to help us to learn about the conditions of mothers giving birth in hospitals and their newborns as well as to find out how to improve the quality of care provided to them. By putting your information together with the information about hundreds of other women that give birth in hospitals across Ghana around this time, we will be able to look for patterns that will tell us whether the new way of working is helpful. We will give presentations and write reports about this, but this will never include your name or any other personal information about you.

**Who will we share your personal information with?**

Your personal data will be collected and processed by the research teams at IFS and OPM. We will share your personal information with Ghana Health Services. In future we will also share an anonymous version of your information with researchers outside the study team.

*Ghana Health Services (GHS):* A few months after we have finished collecting data on everyone taking part in the study, we will send your name, address, and date of birth to GHS and ask them to find your information in their databases. We will not send them your questionnaire answers. GHS might also contact your local community health centre and send us your and your child’s health records. This may for example be postnatal checkups that you may have gone to.

*Microsoft Azure Cloud Services:* Azure are a cloud services platform that IFS uses to store all their information. Azure complies with a number of data security standards (<https://learn.microsoft.com/en-us/azure/compliance/>). Azure acts as a data processor for IFS but does not directly access your personal information.

*Dropbox*: In order to transfer your personal information between DataPivot and the IFS, we will use Dropbox. Before transferring your data to Dropbox, it will be encrypted and protected with a password. Your personal information will be stored on Dropbox only during the transfer. The receiving organisation will delete your information from Dropbox once it has been stored on their own system. Dropbox complies with a number of data security standards: <https://www.dropbox.com/en_GB/business/trust/compliance/certifications-compliance>. Dropbox acts as a data processor but does not directly access your personal information.

*Other researchers*: In the future, we may also publish a version of the analysis dataset that is completely anonymised. This means that we will remove to your name, address, full date of birth and any other detailed information that is likely to identify you. This means that it would not be possible to link this data back to your personal information. The reason for publishing such a dataset is to enable other researchers to analyse and learn from the data.

**What does the law say about using and storing my information?**

UK law (UK GDPR) says that each data controller must have a “lawful basis” for processing and storing your information in the way that we have described.

IFS and OPM’s legal basis for processing information and in this study is *Legitimate Interest (Article 6(1)(f) of the UK GDPR).* Our legitimate interest is research into the best way to support women giving birth and the journey of being a parent of a 0–3-year-old.

By maintaining the IFS archive and allowing approved researchers to access the information in the archive, the IFS is performing a task in the public interest, and this gives the IFS a lawful basis to use personal information.

Certain bits of information about you are known as “Special Category Data” and require more legal protection. This includes things like your ethnicity and wellbeing. This kind of information needs an extra condition for processing. The condition that applies to your data in this study is Article 9 (2) (j) Archiving, research and statistics.

**How long do we keep your information for?**

The data which includes the information that you share in your questionnaires and any administrative records from GHS will be stored for as long as necessary to understand what all the information tells us about service provision to mothers during childbirth and the implications for the mother and child thereafter. The project will start in July 2024. Your information will then be stored for a minimum of 10 years. This is to allow us time to look at the longer-term effects. After 10 years, we will carry out a review to see if there is still useful work that can be done using your data. At any point that we no longer need your data, we will delete it.

The data including your personal information that is stored on the IFS secure server will be stored indefinitely to allow for long term follow up. However, IFS will review every five years to decide whether the information could still be helpful in future research. If it is not, then the information will be deleted.

**Your rights**

You have the right to:

• ask for access to the personal information that we hold about you;

• ask us to correct any personal information that we hold about you which is incorrect, incomplete or inaccurate.

In certain circumstances, you also have the right to:

• ask us to erase the personal information where there is no good reason for us continuing to hold it – please read the information below about the time limits for requesting deletion of your personal information;

• object to us using the personal information for public interest purposes;

• ask us to restrict or suspend the use of the personal information, for example, if you want us to establish its accuracy or our reasons for using it.

**Time limits for deleting your data**

Your survey responses, which contain your personal information, will be kept by the IFS on an ongoing basis and can be deleted any time. However, any data sets that do not include your name and other identifying information for use by other researchers it won’t be possible to delete your information from those archives because we won’t be able to identify you.

If you wish to exercise your rights, please contact us on dataprotectionofficer@ifs.org.uk.

**Who can I speak to if I have any questions?**

You can contact both the Institute for Fiscal Studies (IFS) and OPM by emailing [britta_a@ifs.org.uk](mailto:britta_a@ifs.org.uk) and [Shafique.arif@opml.co.uk](mailto:Shafique.arif@opml.co.uk).

**Other contact details**

You can find information and other contact details of each of the data controllers on their webpages:

Institute for Fiscal Studies: [www.ifs.org.uk](http://www.ifs.org.uk/)

Oxford Policy Management: <https://www.opml.co.uk/>, and the particular project this study falls under, Thrive: <https://www.opml.co.uk/projects/thrive-early-childhood-development>

**Who can I speak to if I want to make a complaint?**

If you want to make a complaint about our use of personal data, please contact the Data Protection Officer at dataprotectionofficer@ifs.org.uk.  Complaints will be dealt with on a case-by-case basis following our complaints procedure.

You can also make a complaint to the Information Commissioner’s Office (ICO) (The UK’s data protection regulator) via their website [https://ico.org.uk](https://ico.org.uk/), by phone 03031231113, or by writing to Information Commissioner’s Office, Wycliffe House, Water Lane, Wilmslow, Cheshire, SK9 5AF

**PART 3: CONSENT**

**Statement of consent/assent**

I certify that I have read the informed consent and privacy notice, or that it was read to me in a language I understand and that my questions have been answered satisfactorily. I understand that I am participating freely and without being forced in any way to do so. I also understand that I can stop participating at any point should I not want to continue, and that this decision will not in any way affect me negatively. I understand that this is a research project whose purpose is not necessarily to benefit me or my child personally in the immediate or short term. I understand that my participation and all others I consent to, will remain confidential.

PLEASE CIRCLE THE APPROPRIATE ANSWER:

1. I confirm that I have read or listened to the Information Sheet and the Privacy Notice, and I have had the opportunity to ask questions. YES/NO
2. As mother/primary caregiver I consent voluntarily to me and my child participating in all described in the Information Sheet and the Privacy Notice. YES/NO
3. I give permission for the study team to access and process the data collected on me and my child in the way described in the Information Sheet and the Privacy Notice. YES/NO
4. (If the mother/primary caregiver is a minor - less than 18 years old, then consent from guardian in addition to mother/primary caregiver:) As the guardian of the mother/primary caregiver, I consent for her, and her child to participate in all described in the Information Sheet and the Privacy Notice. YES/NO
5. (If the mother/primary caregiver is a minor - less than 18 years old, then consent from guardian in addition to mother/primary caregiver:) As the guardian of the mother/primary caregiver, I give permission for the study team to access and process the data collected on the mother/primary caregiver of whom I am the guardian, and her child in the way described in the Information Sheet and the Privacy Notice. YES/NO
6. I have received the contact details of a person to contact if I need to speak to someone about any issues that may arise from this study or make a complaint. YES/NO
7. I agree that the research team will use my data for research in the future and understand that my identifiable data will not be used for any purpose that has not been approved by a relevant research ethics committee. YES/NO
8. I agree that the research team can contact me again in future for follow up questions. YES/NO

**NAME AND SIGNATURE/THUMB STAMP OF THE PARTICIPANT:**

| Name | | Signature or thumb stamp | If no signature/thumb stamp:  Oral consent:  YES/NO |
| --- | --- | --- | --- |
| Participant Name: |  |  |  |
| Witness Name (only if participant cannot read): |  |  |  |

|  |  |  |
| --- | --- | --- |
| Enumerator Name (print) |  | Enumerator Signature |
|  |  |  |
| Date |  | Place |

**INFORMED CONSENT FOR HOSPITAL STAFF**

**Introduction**

Good day, my name is [NAME OF ENUMERATOR]. I have come from DataPivot. DataPivot is working in collaboration with researchers at the Institute for Fiscal Studies and Oxford Policy Management.

**PART 1: INFORMATION SHEET**

**Background and purpose of the research:**

The study will contribute to our understanding on how to improve quality of early childhood service delivery. The focus is on improving the birth process and survival, and the impacts such improvements have on maternal and child outcomes in the longer-term. This will help to have better strategies to deliver and raise Ghanaian children and to boost their skills and intelligence.

**Nature of the research**

We are asking staff working in hospitals across Ghana to take part in this study. This is why you have been chosen. We will talk to these staff like you, as well as mothers who give birth in hospitals. We will also make some observations about what happens in the hospital, particularly in the maternity department. The results of this research will be shared with GHS and the Ministry of Health to understand how they can better design policies to improve the health and development of young children. However, your responses will remain confidential and any data shared with GHS will be anonymised and aggregated.

**Participant involvement:**

We will give you information to help you decide whether you would like to take part in the study. There may be some words that you do not understand. Please ask me to stop as we go through the information, and I will take time to explain.

*What is involved?* Today, we will take some personal information from you, such as your name and contact details. The survey will then cover work experience and conditions, time use, mental health, personality traits, well-being and burnouts, work climate, knowledge, and relationship with other health staff. We will also ask you to play a behavioural game with us and conduct a task with some of your colleagues. We will provide more details on the type of data we aim to collect further below, when we are also explaining how we use and look after your information. Once all the information provided is clear to you, we will ask you for your consent to participate.

*Duration?* We expect today’s interview to take around 45 minutes once consent is provided. We will also ask you for permission to contact you again in the future.

*Right to Refuse or Withdraw:* Participation in this study is voluntary and you may choose not to participate. If you decide not to participate or to withdraw, there will be no repercussions. You can withdraw your consent from the study at any time, either during this survey or after. No data will be submitted without your explicit consent, which is gathered at the beginning and end of the survey. If, in the future, you wish to withdraw your details from the survey or withdraw your consent, you can do so at any time by contacting us at [datapivotgh@gmail.com](mailto:datapivotgh@gmail.com).

*Benefits of taking part in this research:* There are no direct benefits to you from participating in the survey. However, the information you would provide will help us design effective training programs for service delivery to (expecting) parents. Further, by participating in the behavioral games, you have the opportunity to win or donate an estimated 40 cedis. In addition, one of the games will be played in a team and you will win a minimum of 100 cedis from participating and following the rules.

*Disadvantages and/or risks of taking part in the study:* The collection of your information will not put you at risk. All those involved in data collection are aware that if anything untoward happens while they are conducting interviews, either in their interaction with research participants or in relation to the information they are given, they will report it up the chain of command. Such reporting means that the leadership at DataPivot are quickly made aware in order to quickly decide how to mitigate the issue.

*Confidentiality:* All information we collect is strictly confidential and will only ever be used for academic research purposes. Only members of the research team will be able to access information that identifies you and it will only be used for academic research purposes either now or in the future. Not even GHS will know the answers you provide. After removing information that personally identifies you that data might be used by other researchers outside the team. We provide more information about how we handle your personal data below.

*Outcome and feedback:* A summary report of key findings will be made publicly available on the following websites: [www.ifs.org.uk](http://www.ifs.org.uk/), <https://www.opml.co.uk/>, and the Thrive project website: <https://www.opml.co.uk/projects/thrive-early-childhood-development>. We will also send participating staff a summary.

*Funding information:* The research is funded by the Foreign, Commonwealth & Development Office (FCDO), UK, under contract reference PO10087.

*Provision of Information and Consent for participants:* We will give you a copy of this information sheet and the following consent form for you to keep after it has been signed or thumb printed.

**Contact information:**

If you have a question later that you didn't think of now, you can contact DataPivot at datapivotgh@gmail.com. If you want to get further clarification on ethical issues and your rights as participants, you can contact Nana Abena Apatu on 0503539896 or at ethics.research@ghs.gov.gh. If there is any part of this explanation that you do not understand, you should ask before signing. For English speakers, you can also call +44 (0)207 291 4800, and ask for the Principal Investigator of this research: Dr. Britta Augsburg (Associate Director, The Institute for Fiscal Studies) or ask for Zoe Oldfield (Data Protection Officer (DPO), The Institute for Fiscal Studies). You can also contact both on e-mail ([britta_a@ifs.org.uk](mailto:britta_a@ifs.org.uk) and [zoe_o@ifs.org.uk](mailto:zoe_o@ifs.org.uk), respectively).

**PART 2: PRIVACY NOTICE**

Before asking for your consent, we will now read the privacy notice to you. Because the research team is based in the UK, your information is controlled by strict data protection laws. This privacy notice is a legal document and contains some complex language so please ask me if you do not understand something.

**PRIVACY NOTICE FOR HOSPITAL STAFF**

**What is a privacy notice?**

A privacy notice is an important document which tells you how we will use and look after any information that we collect about you. This privacy notice contains all the information that we are required to give you under the UK General Data Protection Regulation (UK GDPR).

**Who are we?**

There are key organisations involved and responsible in the processing and storing of your information. These are the Institute for Fiscal Studies (IFS), Oxford Policy Management (OPM), the University of Cape Coast (UCC), and DataPivot. Our contact details can be found the end of this privacy notice.

**Who is responsible for looking after the information you have about me?**

“Data controller” is a legal phrase, which tells you who makes the decisions about how your information will be used and looked after. The IFS and OPM are the data controllers of the information collected as part of this study. This means that IFS and OPM are independently responsible for processing and looking after your information whilst the data are being collected and analysed by the team.  DataPivot will be responsible for carrying out the interviews with you and transferring the information to us and the University of Cape Coast will be supporting the work that DataPivot does. Because they act only on the instructions of IFS and OPM, DataPivot and UCC are data processors of your information.

**What information do we collect about you?**

The information that we collect, use and store will include personal information about you, including:

- Your name and contact details,
- Information about yourself, such as your age, race or ethnicity,
- Information about your experience, thoughts, feelings, and behavior that you share with us as part of the questionnaires,
- Behavioral games, which may be video recorded and photographed.

**How do we use your information?**

We will use the information you give us to help us to learn about the conditions of mothers giving birth in hospitals and their newborns as well as to find out how to improve the quality of care provided to them. By putting your information together with the information about 375 other hospital staff working across Ghana, we will be able to look for patterns that will tell us whether the new way of working is helpful. We will give presentations and write reports about this, but this will never include your name or any other personal information about you.

**Who will we share your personal information with?**

Your personal data will be collected and processed by the research teams at IFS and OPM.  We will share your personal information with CS Pro. In future we will also share an anonymous version of your information with researchers outside the study team.

*Microsoft Azure Cloud Services:* Azure are a cloud services platform that IFS uses to store all their information. Azure complies with a number of data security standards (<https://learn.microsoft.com/en-us/azure/compliance/>). Azure acts as a data processor for IFS but does not directly access your personal information.

*Dropbox*: In order to transfer your personal information between DataPivot and the IFS, we will use Dropbox. Before transferring your data to Dropbox, it will be encrypted and protected with a password. Your personal information will be stored on Dropbox only during the transfer. The receiving organisation will delete your information from Dropbox once it has been stored on their own system. Dropbox complies with a number of data security standards: <https://www.dropbox.com/en_GB/business/trust/compliance/certifications-compliance>. Dropbox acts as a data processor but does not directly access your personal information.

*Other researchers*: In the future, we may also publish a version of the analysis dataset that is completely anonymised. This means that we will remove to your name, address, full date of birth and any other detailed information that is likely to identify you. This means that it would not be possible to link this data back to your personal information. The reason for publishing such a dataset is to enable other researchers to analyse and learn from the data.

**What does the law say about using and storing my information?**

UK law (UK GDPR) says that each data controller must have a “lawful basis” for processing and storing your information in the way that we have described.

IFS and OPM’s legal basis for processing information and in this study is *Legitimate Interest (Article 6(1)(f) of the UK GDPR).* Our legitimate interest is research into the best way to support women giving birth.

By maintaining the IFS archive and allowing approved researchers to access the information in the archive, the IFS is performing a task in the public interest, and this gives the IFS a lawful basis to use personal information.

Certain bits of information about you are known as “Special Category Data” and require more legal protection. This includes things like your ethnicity and wellbeing. This kind of information needs an extra condition for processing. The condition that applies to your data in this study is Article 9 (2) (j) Archiving, research and statistics.

**How long do we keep your information for?**

The data which includes the information that you share in your questionnaires and any administrative records from GHS will be stored for as long as necessary to understand what all the information tells us about service provision to mothers during childbirth and the implications for the mother and child thereafter. The project will start in July 2024. Your information will then be stored for a minimum of 10 years. This is to allow us time to look at the longer-term effects. After 10 years, we will carry out a review to see if there is still useful work that can be done using your data. At any point that we no longer need your data, we will delete it.

The data including your personal information that is stored on the IFS secure server will be stored indefinitely to allow for long term follow up. However, IFS will review every five years to decide whether the information could still be helpful in future research. If it is not, then the information will be deleted.

**Your rights**

You have the right to:

• ask for access to the personal information that we hold about you;

• ask us to correct any personal information that we hold about you which is incorrect, incomplete or inaccurate.

In certain circumstances, you also have the right to:

• ask us to erase the personal information where there is no good reason for us continuing to hold it – please read the information below about the time limits for requesting deletion of your personal information;

• object to us using the personal information for public interest purposes;

• ask us to restrict or suspend the use of the personal information, for example, if you want us to establish its accuracy or our reasons for using it.

**Time limits for deleting your data**

Your survey responses, which contain your personal information, will be kept by the IFS on an ongoing basis and can be deleted any time. However, any data sets that do not include your name and other identifying information for use by other researchers it won’t be possible to delete your information from those archives because we won’t be able to identify you.

If you wish to exercise your rights, please contact us on dataprotectionofficer@ifs.org.uk.

**Who can I speak to if I have any questions?**

You can contact both the Institute for Fiscal Studies (IFS) and OPM by emailing the research team at [britta_a@ifs.org.uk](mailto:britta_a@ifs.org.uk) or shafique.arif@opml.co.uk.

**Other contact details**

You can find information and contact details of each of the data controllers on their webpages:

Institute for Fiscal Studies: [www.ifs.org.uk](http://www.ifs.org.uk/)

Oxford Policy Management: <https://www.opml.co.uk/>, and the particular project this study falls under, Thrive: <https://www.opml.co.uk/projects/thrive-early-childhood-development>

**Who can I speak to if I want to make a complaint?**

If you want to make a complaint about our use of personal data, please contact the Data Protection Officer at dataprotectionofficer@ifs.org.uk.  Complaints will be dealt with on a case-by-case basis following our complaints procedure.

You can also make a complaint to the Information Commissioner’s Office (ICO) (The UK’s data protection regulator) via their website [https://ico.org.uk](https://ico.org.uk/), by phone 03031231113, or by writing to Information Commissioner’s Office, Wycliffe House, Water Lane, Wilmslow, Cheshire, SK9 5AF

**PART 3: CONSENT**

**Statement of consent/assent**

I certify that I have read the informed consent and privacy notice, or that it was read to me in a language I understand and that my questions have been answered satisfactorily. I understand that I am participating freely and without being forced in any way to do so. I also understand that I can stop participating at any point should I not want to continue, and that this decision will not in any way affect me negatively. I understand that this is a research project whose purpose is not necessarily to benefit me personally in the immediate or short term. I understand that my participation and all others I consent to, will remain confidential.

PLEASE CIRCLE THE APPROPRIATE ANSWER:

1. I confirm that I have read or listened to the Information Sheet and the Privacy Notice, and I have had the opportunity to ask questions. YES/NO
2. As hospital staff I consent voluntarily to me participating in all described in the Information Sheet and the Privacy Notice. YES/NO
3. I give permission for the study team to access and process the data collected on me in the way described in the Information Sheet and the Privacy Notice. YES/NO
4. I have received the contact details of a person to contact if I need to speak to someone about any issues that may arise from this study or make a complaint. YES/NO
5. I consent to my name, address, and date of birth, without my questionnaire answers, being shared with Ghana Health Services to allow my information to be linked to my administrative records so that researchers can use this information alongside my questionnaire answers. YES/NO
6. I agree that the research team will use my data for research in the future and understand that my identifiable data will not be used for any purpose that has not been approved by a relevant research ethics committee. YES/NO
7. I agree that the research team can contact me again in future for follow up questions. YES/NO
8. You will be later asked to participate in games with your team. During these games, and only during these games, you may be video recorded as well as photographed. The photographs and video recording may only happen if you consent to it in this form and you will not be recorded, filmed or photographed against your will. The researcher team will only ever use those for academic and dissemination purposes.
9. I consent to me to be video-recorded. YES/NO
10. I consent to me to be photographed. YES/NO

**NAME AND SIGNATURE/THUMB STAMP OF THE PARTICIPANT:**

| Name | | Signature or thumb stamp | If no signature/thumb stamp:  Oral consent:  YES/NO |
| --- | --- | --- | --- |
| Participant Name: |  |  |  |
| Witness Name (only if participant cannot read): |  |  |  |

|  |  |  |
| --- | --- | --- |
| Enumerator Name (print) |  | Enumerator Signature |
|  |  |  |
| Date |  | Place |

**S.2 Data Statement:**

**WHO Data set**

| Primary Registry and Trial Identifying Number | <https://www.isrctn.com/ISRCTN15629047> |
| --- | --- |
| Date of Registration in Primary Registry | 04/11/2024 |
| Secondary Identifying Numbers | N/A |
| Source(s) of Monetary or Material Support | Foreign, Commonwealth & Development Office, UK;  Economic and Social Resource Council, UK;  Grand Challenges Canada |
| Primary Sponsor | The Institute for Fiscal Studies |
| Secondary Sponsor | N/A |
| Contact for Public Queries | Britta Augsbury: [britta_a@ifs.org.uk](mailto:britta_a@ifs.org.uk)  The Institute for Fiscal Studies  Tel: 020 7291 4800  7 Ridgmount Street  London  WC1E 7AE |
| Contact for Scientific Queries | Britta Augsbury: [britta_a@ifs.org.uk](mailto:britta_a@ifs.org.uk)  The Institute for Fiscal Studies  Tel: 020 7291 4800  7 Ridgmount Street  London  WC1E 7AE |
| Public Title | Evaluating an obstetric triage training programme at scale in Ghana |
| Scientific Title | Enhancing Maternal and Newborn Outcomes in Ghana: A Comprehensive Randomized Controlled Trial Evaluation of Obstetric Triage Effectiveness and Midwives Training |
| Countries of Recruitment | Ghana |
| Health Condition(s) or Problem(s) Studied | quality of service during labour and delivery, as well as on maternal and neonatal survival, and on neonatal health outcomes; clinical knowledge and midwives’ attitudes |
| Interventions | The Obstetric Triage Implementation Package (OTIP), a 1-week on-site training on clinical knowledge to quickly assess and prioritize the care of pregnant women, with the goal of promptly and accurately identifying the severity of a patient’s condition, determine the appropriate level of care, and ensure that those with the most critical needs receive immediate attention. |
| Key Inclusion and Exclusion Criteria | 1. A midwife working in a study hospital, including all selected as champions 2. A patient (mother) who gave birth in the hospital within the two months prior to the survey. |
| Study Type | Interventional, open label cluster randomized control trial. |
| Date of First Enrolment | 09/09/2024 |
| Sample Size | 750 midwives  1,250 paatients (mothers) |
| Recruitment Status | Incomplete |
| Primary Outcome | **Process variables**: Representing the actual medical care received by mothers and their new-borns (e.g., time until initial assessment; assessment received upon arrival; doctor intervened if complications during labour and delivery at the hospital; postnatal checks).  **Maternal and neonatal outcome variables**: Including the health of pregnant women and new-borns (e.g. maternal and neonatal mortality; complications during labour and delivery at the hospital; APGAR scores). |
| Key Secondary Outcomes | **Hospital staff outcomes**: Such as improved knowledge and midwives’ attitudes capturing their perceptions of autonomy, empowerment and motivation. |
| Ethics Review | Study protocols have been approved by Ghana Health Service Ethical Review Committee (GHS-ERC: 022/05/24). |
| Completion date | December 2025 |
| Summary Results | n/a as trial incomplete |
| IPD sharing statement | The data collected in the study will be publicly distributed along with critical documents (ie, protocols and questionnaires) following the publication of the primary results from the trials, which is expected to be within 24 months of the final data collection date. |

**S.3 Study locations:**
